# A Case of Comprehensive Interventional Therapy for Bronchial Thromboembolism Caused by Hemoptysis

**DOI:** 10.1002/rcr2.70477

**Published:** 2026-01-19

**Authors:** Qiangmao Wang, Zhanwei Ji, Mingyue Shi

**Affiliations:** ^1^ Jinzhou People's Hospital Shijiazhuang Hebei P. R. China; ^2^ College of Pulmonary & Critical Care Medicine, 8th Medical Centre, Chinese PLA General Hospital Beijing P. R. China

**Keywords:** bronchial artery embolization, bronchial thromboembolism, cryotherapy, hemoptysis, interventional therapy

## Abstract

Massive hemoptysis from dual bronchial artery malformations necessitated emergent embolization. Extensive jelly‐like endobronchial clots caused complete lobar collapse refractory to suction. We used cryoextraction to achieve rapid en‐bloc removal of arborizing casts with prompt radiographic resolution.

## Graphical abstract

We report the case of a patient with massive hemoptysis from dual bronchial artery malformations that necessitated emergent embolization. Extensive jelly‐like endobronchial clots caused complete lobar collapse refractory to suction. We used cryoextraction to achieve rapid, en‐bloc removal of arborizing casts with prompt radiographic resolution.

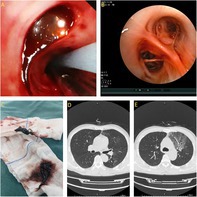

A 52 year old man presented with a 1 day history of cough and hemoptysis. The total volume of expectorated blood was approximately 200 mL. Due to the large volume of bleeding and oxygen saturation decreasing, the patient underwent emergent bronchial artery embolization under Digital Subtraction Angiography (DSA). Angiography revealed a dilated and tortuous left main bronchial artery (Figure 1A), as well as a right‐sided bronchial‐to‐pulmonary arterial fistula (Figure 1B); both lesions were embolized with polyvinyl alcohol (PVA) particles. Post‐procedure, the patient had no further hemoptysis, but oxygen saturation remained low. Computed tomography (CT) of the thorax showed the consolidation of the left upper lobe with obstruction of the segmental and subsegmental bronchi in both the left upper and lower lobes (Figure 2A,B). Fibreoptic bronchoscopy showed the left upper lobe was completely obstructed by jelly‐like thrombi that could not be removed despite repeated suction under negative pressure (Figure 2C,D). We subsequently applied cryotherapy. After cryo‐adhesion at the thrombus sites, segmental thrombi were extracted en bloc (Figure 3A,B); the retrieved casts exhibited a branching, arborizing morphology (Figure 3C). Post‐procedure chest CT confirmed patency of all left‐sided bronchial divisions and showed resolution of the previous consolidation in the left upper lobe (Figure 3D,E).

**FIGURE 1:**
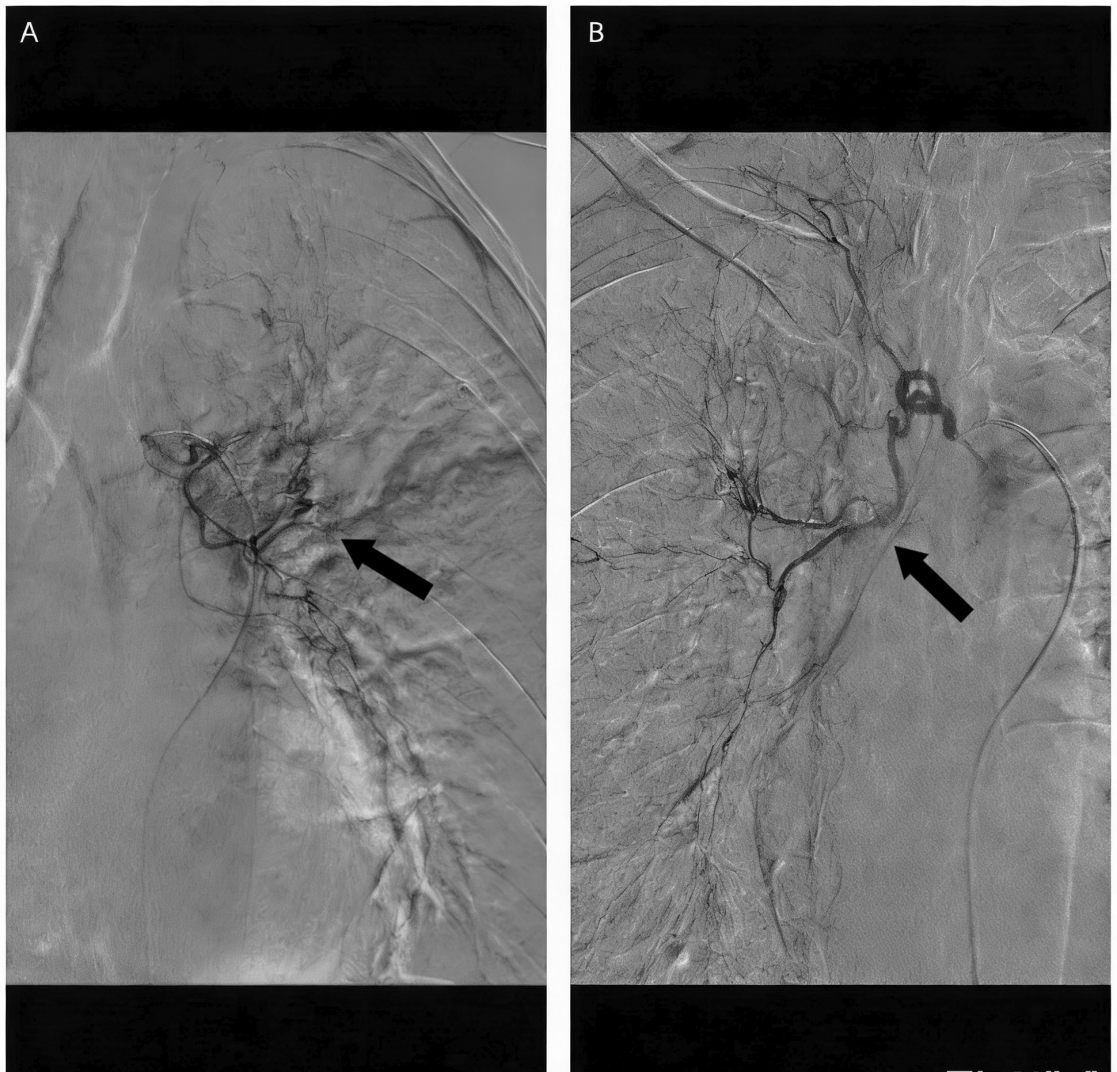
Angiography revealed a dilated and tortuous left main bronchial artery (A black arrows); right‐sided bronchial‐to‐pulmonary arterial fistula (B black arrows).

**FIGURE 2:**
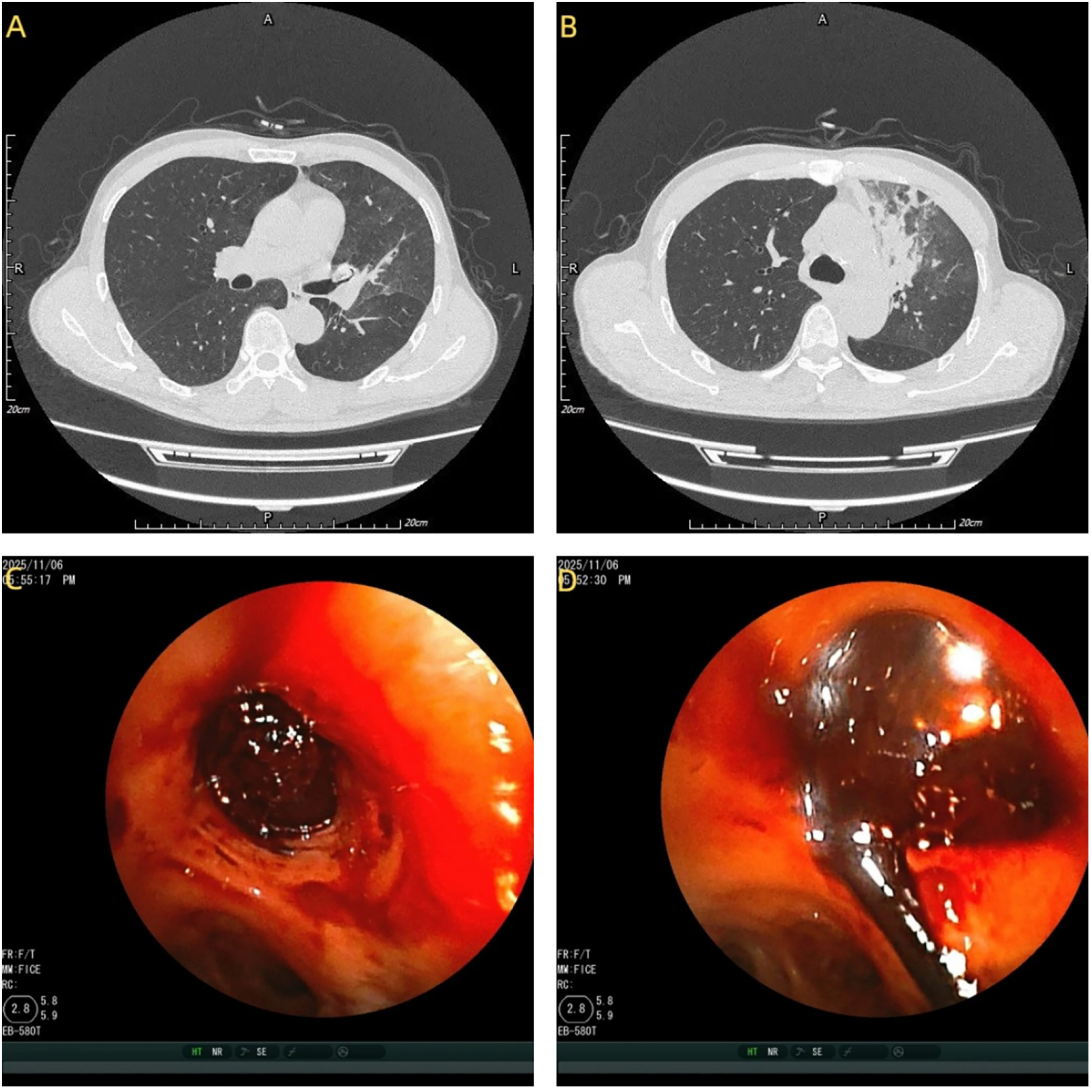
(A, B) CT of the thorax showed the consolidation of the left upper lobe with obstruction of the segmental and subsegmental bronchi in both the left upper and lower lobes; (C, D) fibreoptic bronchoscopy showed the left upper lobe was completely obstructed by jelly‐like thrombi.

**FIGURE 3:**
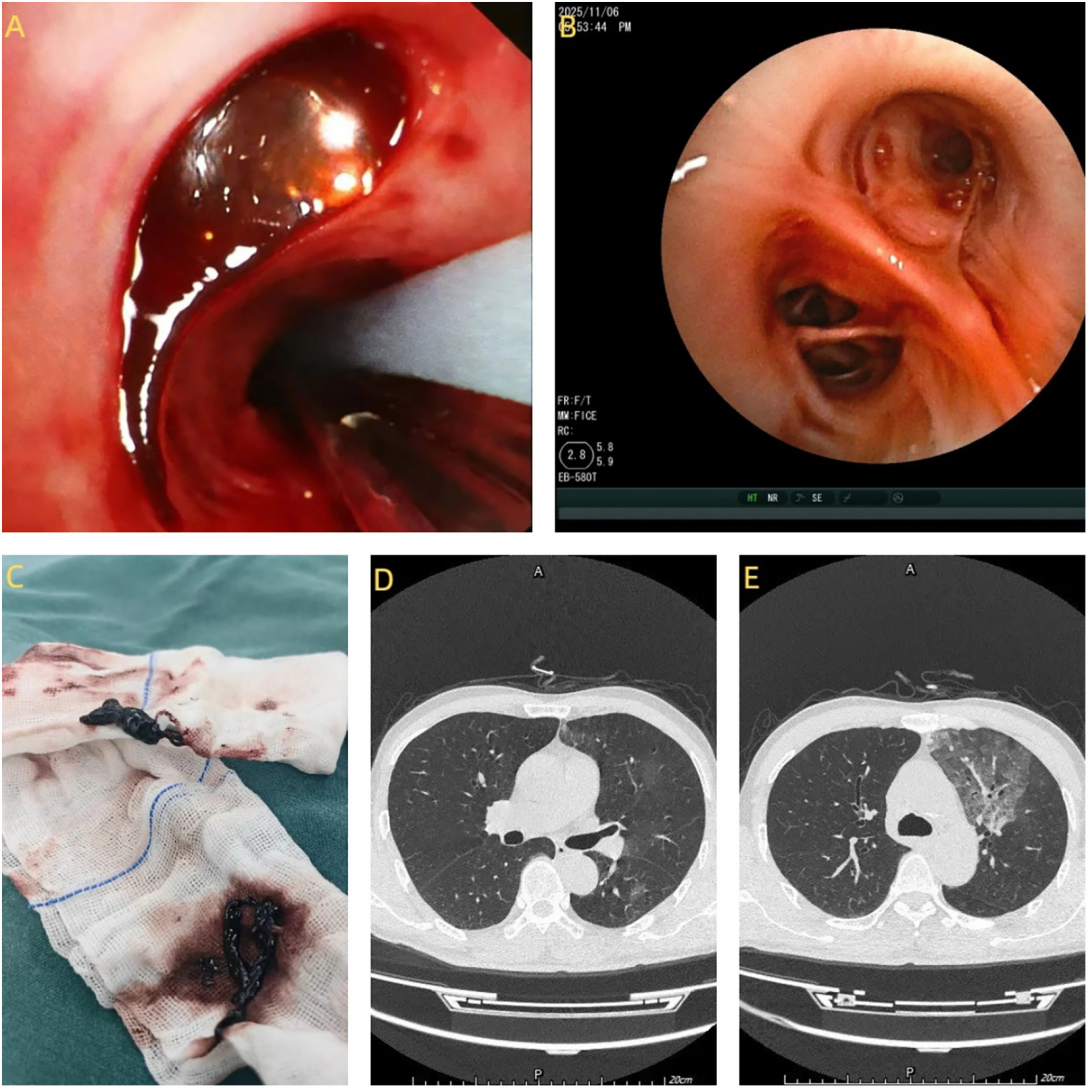
(A) Cryotherapy under the bronchoscopy; (B) the left upper lobes after treatment; (C) the retrieved casts exhibited a branching, arborizing morphology; (D, E) CT of the thorax post‐procedure.

Bronchial artery malformations typically present with hemoptysis as the initial symptom. And the bronchial artery‐pulmonary artery fistulas are particularly difficult to diagnose. Bronchial arteriography followed by embolization is the definitive therapeutic strategy for such vascular anomalies [1, 2]. In our case, massive hemoptysis led to extensive endobronchial thrombotic occlusion that could not be removed by conventional bronchoscopic suction. This manifestation was rare [3]. We employed cryoextraction to achieve complete clearance of the obstructing thrombi, resulting in favourable clinical and radiological outcomes.

## Author Contributions

All of the authors contributed to the writing, review and final approval of the manuscript.

## Funding

The authors have nothing to report.

## Consent

The authors declare that written informed consent was obtained for the publication of this manuscript and accompanying images using the consent form provided by the Journal.

## Conflicts of Interest

The authors declare no conflicts of interest.

## Data Availability

The data that support the findings of this study are available from the corresponding author upon reasonable request.
